# Novel Electroactive Mineralized Polyacrylonitrile/PEDOT:PSS Electrospun Nanofibers for Bone Repair Applications

**DOI:** 10.3390/ijms241713203

**Published:** 2023-08-25

**Authors:** Frederico Barbosa, Fábio F. F. Garrudo, Ana C. Marques, Joaquim M. S. Cabral, Jorge Morgado, Frederico Castelo Ferreira, João C. Silva

**Affiliations:** 1 Department of Bioengineering and iBB—Institute for Bioengineering and Biosciences, Instituto Superior Técnico, Universidade de Lisboa, Av. Rovisco Pais, 1049-001 Lisboa, Portugal; frederico.porto@tecnico.ulisboa.pt (F.B.); fabio.garrudo@tecnico.ulisboa.pt (F.F.F.G.); joaquim.cabral@tecnico.ulisboa.pt (J.M.S.C.); 2 Associate Laboratory i4HB—Institute for Health and Bioeconomy, Instituto Superior Técnico, Universidade de Lisboa, Av. Rovisco Pais, 1049-001 Lisboa, Portugal; 3 Department of Bioengineering and Instituto de Telecomunicações, Instituto Superior Técnico, Universidade de Lisboa, Av. Rovisco Pais, 1049-001 Lisboa, Portugal; jmfmorgado@tecnico.ulisboa.pt; 4 Departament of Chemical Engineering and CERENA—Center for Natural Resources and the Environment, Instituto Superior Técnico, Universidade de Lisboa, Av. Rovisco Pais, 1049-001 Lisboa, Portugal; ana.marques@tecnico.ulisboa.pt

**Keywords:** bone tissue engineering, electrospinning, PAN, PEDOT:PSS, mesenchymal stem/stromal cells, mineralization, osteogenic differentiation

## Abstract

Bone defect repair remains a critical challenge in current orthopedic clinical practice, as the available therapeutic strategies only offer suboptimal outcomes. Therefore, bone tissue engineering (BTE) approaches, involving the development of biomimetic implantable scaffolds combined with osteoprogenitor cells and native-like physical stimuli, are gaining widespread interest. Electrical stimulation (ES)-based therapies have been found to actively promote bone growth and osteogenesis in both in vivo and in vitro settings. Thus, the combination of electroactive scaffolds comprising conductive biomaterials and ES holds significant promise in improving the effectiveness of BTE for clinical applications. The aim of this study was to develop electroconductive polyacrylonitrile/poly(3,4-ethylenedioxythiophene):polystyrene sulfonate (PAN/PEDOT:PSS) nanofibers via electrospinning, which are capable of emulating the native tissue’s fibrous extracellular matrix (ECM) and providing a platform for the delivery of exogenous ES. The resulting nanofibers were successfully functionalized with apatite-like structures to mimic the inorganic phase of the bone ECM. The conductive electrospun scaffolds presented nanoscale fiber diameters akin to those of collagen fibrils and displayed bone-like conductivity. PEDOT:PSS incorporation was shown to significantly promote scaffold mineralization in vitro. The mineralized electroconductive nanofibers demonstrated improved biological performance as observed by the significantly enhanced proliferation of both human osteoblast-like MG-63 cells and human bone marrow-derived mesenchymal stem/stromal cells (hBM-MSCs). Moreover, mineralized PAN/PEDOT:PSS nanofibers up-regulated bone marker genes expression levels of hBM-MSCs undergoing osteogenic differentiation, highlighting their potential as electroactive biomimetic BTE scaffolds for innovative bone defect repair strategies.

## 1. Introduction

Bone defects are associated with the significant localized loss of bone tissue as a result of high energy trauma, infection (osteomyelitis), congenital anomalies or malignancy (tumor resection) [1]. They often require clinical intervention in order to properly fill the damaged area, particularly in the case of critical-sized bone defects, i.e., defect length exceeding 2–2.5 times the diaphyseal diameter of the affected bone, for which spontaneous native bone healing becomes inefficient [2,3]. Autologous bone grafts, usually harvested from the iliac crest of the patients, have become the gold-standard treatment for these types of conditions because of their combined osteogenic, osteoinductive and osteoconductive potential and reduced immunogenic response. Important drawbacks of this therapeutic approach include donor site morbidity, the need for multiple surgical interventions, the potential resorption of the implanted graft and the reduced availability of transplantable bone tissue with adequate mechanical properties [1,4,5]. Moreover, other alternatives such as bone allografts, obtained from available donors, also present meaningful disadvantages such as reduced availability, potential disease transmission, risk of graft rejection and inconsistent donor-dependent mechanical and osteoinductive properties of the grafts [5,6]. For this reason, bone tissue engineering (BTE) strategies currently being investigated as long-term solutions for critical-sized bone defects include the design and fabrication of biocompatible customizable synthetic grafts capable of mimicking the extracellular matrix (ECM) of the native bone tissue and assisting the regeneration process [7,8].

Different electrical stimulation (ES)-based therapies have been explored over the past decades as a complement to standard bone defect repair techniques with positive outcomes in the osseointegration of implanted grafts as well as in the formation of neobone tissue [9,10,11,12]. ES-based strategies have yielded positive results in both experimental and clinical settings. The application of physiological-like low-voltage ES in vitro has been found to boost cell adhesion to biocompatible scaffolds as well as cell migration, proliferation and osteogenic differentiation [11,13,14]. Studies conducted on different animal models reported successful localized bone growth and the up-regulated expression of key growth factors pertaining to osteogenesis in response to ES [11,15,16,17].

Electroactive implantable scaffolds comprising conductive biomaterials are currently under development to support ES-based therapies [18]. Specifically, these structures could be used to direct the electrical stimuli applied to the damaged site, potentially increasing the effectiveness of the regeneration process [19,20]. Additionally, electroconductive scaffolds could be used as bone electrodes to improve cell interaction and reduce the magnitude of foreign body response by the patient’s immune system. This strategy has the potential to avoid/reduce the formation of a fibrotic capsule around the electrodes, which hinders their function and results in complete rejection by the body [21,22].

Various conductive biomaterials have been applied for developing electroconductive scaffolds in the context of BTE. These materials can be essentially categorized into metals, carbon allotropes and conductive polymers [23,24,25]. The excellent conductivity of both metals and different carbon-based allotropes has led to their increasing use in BTE. Gold and silver nanoparticles have been incorporated into the macrostructure of BTE scaffolds (as conductive additives) to improve the mechanical properties and conductivity of the resulting constructs [26,27,28]. The reported osteogenic potential of graphene and carbon nanotubes (CNTs) has also contributed to their relative widespread use in BTE strategies [29,30]. However, the cytotoxicity of these materials continues to limit their potential clinical applications [25,28,29,31]. Conductive polymers (CPs) combine the excellent electrical properties of metals and semiconductors with the versatility, ease of synthesis and flexibility commonly attributed to organic polymers [23,25]. Poly(3,4-ethylenedioxythiophene):polystyrene sulfonate (PEDOT:PSS) is a biocompatible CP comprising a positively charged PEDOT phase and a negatively charged PSS phase. The high conductivity of PEDOT:PSS, combined with its enhanced electrochemical stability, high biocompatibility and easiness of processing when compared with other CPs, has motivated its increasing use in several BTE strategies [32,33].

Synthetic grafts should mimic the structural features of the tissue they are intended to emulate. Since collagen fibers represent a significant component of the ECM of multiple tissues, including bone, the development of fibrous scaffolds has been gaining interest. Electrospinning is the most widely applied technique to produce fibers with small diameters ranging from a few nanometers to micrometers [24,34]. Several studies have described the production of electroconductive electrospun nanofibers by applying the conductive material as a fiber coating. Other works have reported the blending of a non-conductive polymer, used as a carrier, with the conductive material prior to electrospinning in order to improve the spinnability of the casting solution [35,36,37].

Overall, the production of electroconductive fibrous scaffolds in the context of BTE has remained largely unexplored [24]. However, there are several examples of production of electroconductive and electrospun fibers for other targets in tissue engineering. Magaz et al. (2020) reported the fabrication of PEDOT:PSS-coated silk fibroin nanofibers that were able to support NG108-15 neuronal cell proliferation and differentiation [38]. In another study, Abedi et al. (2019) developed polyvinyl alcohol (PVA)-reinforced chitosan/PEDOT:PSS nanofibers with varying PEDOT:PSS concentrations, which displayed electrical and mechanical properties akin to those of the cardiac tissue [39]. Such electroconductive nanofibrous scaffolds could also be applied in BTE applications. 

In this study, we describe the fabrication of novel PEDOT:PSS nanofibers via electrospinning capable of mimicking key features of the bone’s ECM and providing a platform for the application of exogenous ES. The scaffolds were prepared by blending the CP with non-conductive polyacrylonitrile (PAN), used as a carrier polymer, adapting a novel protocol previously described by Garrudo et al. (2023) [40]. The resulting constructs were annealed and dipped in sulfuric acid in order to enhance the electroconductivity of the nanofibers. The scaffolds were also mineralized in Simulated Body Fluid (SBF) to replicate the inorganic phase of the bone ECM. The obtained PAN/PEDOT:PSS nanofibrous scaffolds were characterized in terms of their structural, chemical and electrical properties. Finally, the mineralized electroactive scaffolds were cultured with a human osteoblast-like cell line and human bone marrow-derived mesenchymal stem/stromal cells (hBM-MSCs) in order to assess their ability to support cell growth and osteogenic differentiation in comparison to their non-mineralized and non-conductive counterparts. The scaffolds generated in this work are among the first PEDOT:PSS-based electrospun nanofibers reported for BTE, and they are also one of the few electroconductive nanofibers that have been produced by blending PEDOT:PSS with a non-conductive carrier polymer, instead of using it as a fiber coating (through dip coating, chemical vapor deposition). This strategy allows a more homogenous distribution of the CP and a more efficient transfer of electrical signals [37]. The use of hydrophobic PAN as a carrier polymer also obliviates the need for often cytotoxic crosslinking agents (e.g., GOPS, glutaraldehyde) to prevent PEDOT:PSS dissolution [39,41]. Importantly, the nanofibers fabricated in this study are also, to the best of our knowledge, the first mineralized electrospun PEDOT:PSS-based scaffolds for BTE applications.

## 2. Results and Discussion

### 2.1. Production and Physicochemical Characterization of PAN/PEDOT:PSS Nanofibers before Post-Processing

**Figure 1:**
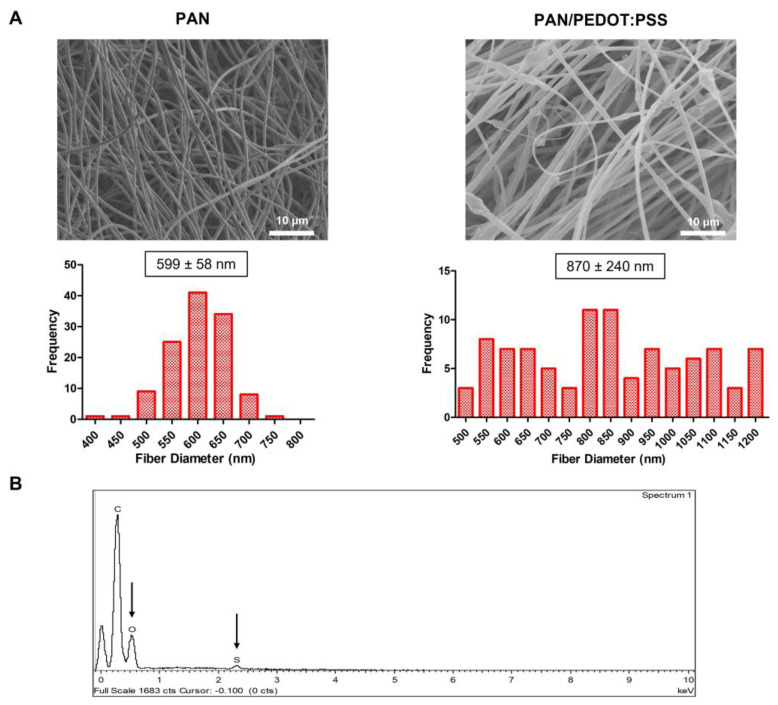
SEM micrographs ((**A**), **top**) and respective fiber diameter distribution histograms ((**A**), **bottom**) of the electrospun PAN and PAN/PEDOT:PSS nanofibers prior to HAT treatment. Scale bar: 10 μm. Elemental composition analysis (EDX) (**B**) of the generated composite PAN/PEDOT:PSS scaffolds prior to HAT treatment. The PEDOT:PSS-related sulfur and oxygen peaks detected in the EDX spectra of the sample are highlighted with a black arrow.

**Figure 2:**
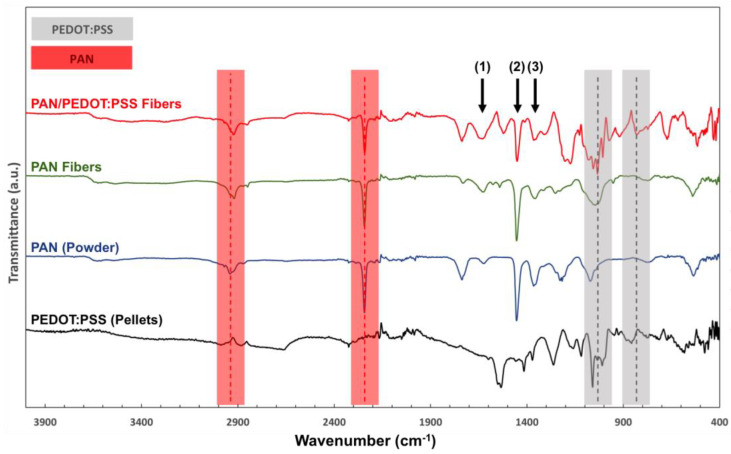
FTIR analysis of PEDOT:PSS pellets, PAN powder and PAN and PAN/PEDOT:PSS nanofibers. PAN-related IR peaks (2242 cm^−1^ and 2938 cm^−1^) are highlighted in red. PEDOT:PSS-related IR peaks (832 cm^−1^ and 1032 cm^−1^) are highlighted in gray. Overlapping PAN- and PEDOT:PSS-related IR peaks are identified with black arrows: (1) 1623 cm^−1^ and 1640 cm^−1^, (2) 1453 cm^−1^ and 1455 cm^−1^ and (3) 1357 cm^−1^ and 1350 cm^−1^, for PAN and PEDOT:PSS, respectively.

PAN and PAN/PEDOT:PSS nanofibers were successfully produced by electrospinning using previously optimized operational parameters [40]. Scanning electron microscopy (SEM) and Energy Dispersive X-ray (EDX) analyses of the scaffolds were conducted to assess fiber morphology and composition, respectively (Figure 1). As shown in Figure 1A, a slight increase in fiber average diameter was observed with the addition of PEDOT:PSS to the electrospinning solutions. This result is in line with the ones reported by Lim et al. (2023), in which higher fiber diameters were obtained by increasing the amount of PEDOT:PSS added to non-conductive polyvinylidene fluoride (PVDF) electrospun fibers [42]. The obtained fibers also exhibited a smooth surface and an overall reduced number of artifacts (bead-free). A more heterogenous set of nanofibers, in terms of fiber diameter, was obtained for the PAN/PEDOT:PSS scaffolds when compared with the control fibrous mats composed of PAN alone. This may be related to an augmented jet instability during electrospinning due to the higher conductivity of the PAN/PEDOT:PSS solution in comparison to the PAN solution [43,44]. The presence of PEDOT:PSS in the fiber composition was confirmed with the detection of sulfur and oxygen in the EDX spectra of the composite scaffolds (Figure 1B). No nitrogen peaks, expected due to the presence of PAN, were detected in the EDX spectra of the nanofibers, which likely occurs because the radiation emitted by nitrogen atoms is being absorbed by the beryllium window of the spectrometer and, consequently, hindering their detection [45]. The presence of the non-conductive PAN polymer was later confirmed by FTIR analysis (Figure 2).

The combination of PEDOT:PSS and PAN led to a meaningful improvement in the wettability of the scaffolds (Supplementary Material, Figure S1). The control PAN nanofibers were found to be slightly hydrophobic, presenting a contact angle above 90° (95.2 ± 13.1°). The PAN/PEDOT:PSS scaffolds, on the other hand, were extremely hydrophilic, quickly adsorbing the water droplets being deposited on their surface (sessile drop technique), thus making contact angle measurements unfeasible. The hydrophilic nature of PSS and potential segregation of this polymer to the surface of the fibers could be potential justifications for the results observed [32]. A similar trend in contact angle was reported by Babaie et al. (2020) for PVA-PEDOT:PSS blends [46].

The chemical properties of the scaffolds were characterized by FTIR analysis (Figure 2). For both PAN and PAN/PEDOT:PSS nanofibers, infrared (IR) peaks commonly attributed to PAN could be identified in the collected spectra, namely at ca. 2242 cm^−1^ (-C≡N stretching) and 2938 cm^−1^ (−CH_2_ stretching) [47,48]. Moreover, two major IR peaks assigned to PEDOT:PSS were also identified on the FTIR spectra of the composite PAN/PEDOT:PSS fibers, further corroborating the presence of the CP on the structure of those scaffolds: 832 cm^−1^ (−C−S−C stretching, thiophene ring) and 1032 cm^−1^ (−SO_3_ symmetric stretching, PSS) [49,50]. Additional IR peaks related to the vibrations of the aliphatic CH groups of PAN (1357 cm^−1^ and 1453 cm^−1^) and the thiophene rings, −C−C− (C_b_-C_b_) and –C=C– (C_a_-C_b_) of PEDOT (1350 cm^−1^ and 1455 cm^−1^, respectively), were also identified, although their individual contributions to the FTIR spectra of the PAN/PEDOT:PSS scaffolds could not be assessed due to peak overlap [47,50]. Similarly, two additional overlapping IR peaks assigned to the stretching of the amide NH_2_ groups of PAN (1623 cm^−1^) and the asymmetric stretching of the –C=C– groups from both PEDOT and the phenyl side group of PSS (1640 cm^−1^) were also detected [47,50,51].

Four-contact conductivity measurements of both PAN and PAN/PEDOT:PSS nanofibers were performed in order to evaluate the electrical properties of the as-spun fibrous scaffolds. The PAN/PEDOT:PSS fibers were found to be non-conductive prior to being annealed and doped in sulfuric acid (HAT treatment). This is attributed to the larger amount of non-conductive PAN in the fibers with respect to the DMSO-doped PEDOT:PSS (PAN content, by weight, is more than three times the PEDOT:PSS).

### 2.2. Physicochemical Characterization of HAT-PAN/PEDOT:PSS Fibers

Various strategies have been developed to improve the electroconductivity of PEDOT:PSS with varying degrees of success [32,33,51,52]. These can be divided into heat treatments, light treatments and treatments with organic solvents (e.g., DMSO, methanol), with the latter strategy attracting the most attention [53]. In particular, the post-treatment of PEDOT:PSS with strong acids has yielded the highest increase in electrical conductivity, reaching maximum conductivity of 4380 S cm^−1^ (four thousand orders of magnitude improvement) for PEDOT:PSS films upon treatment with sulfuric acid, as reported by Kim et al. (2013) [54]. The authors of this study hypothesized that the high electrical conductivity obtained is a result of the stabilization (by using highly concentrated sulfuric acid) of the positively (PEDOT) and negatively (PSS) charged phases of PEDOT:PSS through the production of ions (HSO_4_^-^ and H_3_SO_4_^+^) by autoprotolysis [53,54]. There is also a selective and extensive removal of PSS, accompanied by significant structural rearrangements of the PEDOT:PSS polymeric chains, as high-density and highly ordered PEDOT:PSS fibrils are formed, improving both the (i) charge carrier concentration, but not the oxidation state of PEDOT, ruling out a charge transfer doping of PEDOT and (ii) mobility, consequently increasing considerably the conductivity of the CP [53]. It should be mentioned that though the term “doping” is widely used, one must distinguish the cause of conductivity increase between charge transfer doping (which would imply an increase in the PEDOT oxidation state) and secondary doping, a process by which the additives improve the conductivity via modifications of phase separation and/or conformational changes. Electroconductivity improvements of PEDOT:PSS have been mostly the result of the implementation of secondary doping strategies, which in cases of post-treatment with sulfuric acid, involves also the selective removal of the electronically insulating PSS.

In order to emulate this process and improve fiber conductivity, the electrospun fibrous scaffolds were treated with sulfuric acid (HAT treatment). The PAN/PEDOT:PSS fibers were first annealed at 210 °C for 24 h to stabilize the PAN structure by inducing cyclization (i.e., formation of aromatic groups) and crosslinking between the PAN chains. This annealing step aims to obtain fibers with increased resistance and chemical stability and, therefore, the ability to endure sulfuric acid aggression [55,56,57]. After being annealed, the nanofibers were then immersed in sulfuric acid for 24 h, followed by thermal processing to improve sulfuric acid penetrability and maximize its effects on PEDOT:PSS.

**Figure 3:**
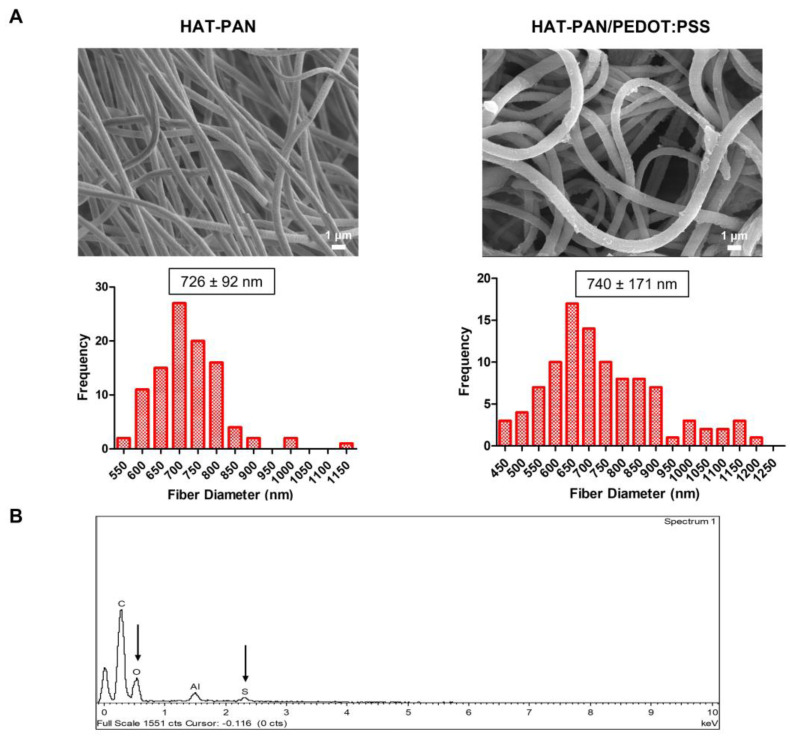
SEM micrographs ((**A**), **top**) and respective fiber diameter distribution histograms ((**A**), **bottom**) of the electrospun HAT-PAN and HAT-PAN/PEDOT:PSS nanofibers. Scale bar: 1 μm. EDX analysis (**B**) of the generated composite HAT-PAN/PEDOT:PSS scaffolds. The PEDOT:PSS-related sulfur and oxygen peaks detected in the EDX spectra of the sample are highlighted with a black arrow.

**Figure 4:**
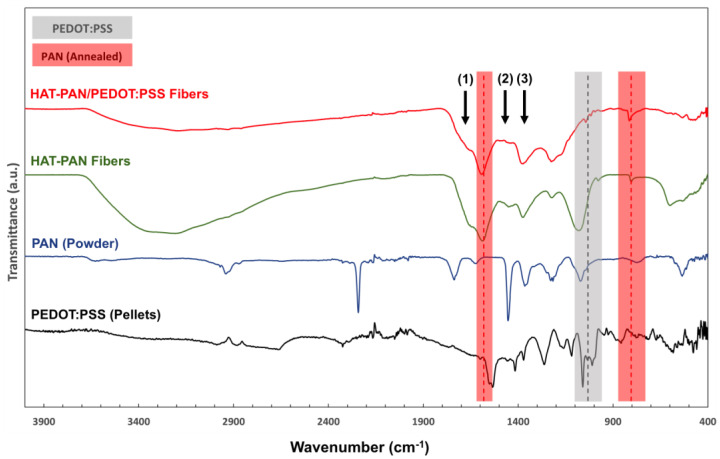
FTIR analysis of PEDOT:PSS pellets, PAN powder and HAT-PAN and HAT-PAN/PEDOT:PSS nanofibers. A PEDOT:PSS-related IR peak (1032 cm^−1^) is highlighted in gray. Two characteristic annealed PAN-related IR peaks (805 cm^−1^ and 1582 cm^−1^) are highlighted in red. Overlapping PAN- and PEDOT:PSS-related IR peaks are identified with black arrows: (1) 1623 cm^−1^ and 1640 cm^−1^, (2) 1453 cm^−1^ and 1455 cm^−1^ and (3) 1357 cm^−1^ and 1350 cm^−1^, for PAN and PEDOT:PSS, respectively.

The resulting HAT-treated PAN (HAT-PAN) and PAN/PEDOT:PSS (HAT-PAN/PEDOT:PSS) nanofibers were imaged with SEM, and their elemental composition was assessed by EDX analysis (Figure 3). As shown in Figure 3A, the scaffolds were able to preserve their fibrous architecture after being immersed in sulfuric acid. A small average diameter decrease was registered for the HAT-PAN/PEDOT:PSS nanofibers, which is likely the result of the selective removal of PSS that occurs upon PEDOT:PSS treatment with sulfuric acid and was later confirmed with FTIR analysis (Figure 4) [58]. On the other hand, a more moderate increase in diameter was observed for the HAT-PAN scaffolds. This could be attributed to an increase in water content of the scaffolds as a result of the sulfuric acid treatment, in which sulfate ions, with a strong hydrating effect, enter the fibers and increase their water uptake [59]. The increased water content of the HAT-PAN scaffolds would also explain the more significant presence of hydroxyl groups (IR range of 3000–3700 cm^−1^) that was detected for the HAT-PAN fibers with FTIR (Figure 4) [60]. No significant changes in the topography of the fibers were observed after HAT treatment. Through EDX analysis, PEDOT:PSS-related sulfur and oxygen were once again identified, confirming the presence of the CP in the structure of the nanofibers after doping (Figure 3B). Similarly to the previous observations, no nitrogen peak was detected.

The HAT treatment did not have any noticeable negative impact on the wettability of the scaffolds (Supplementary Material, Figure S1). A high wettability of the HAT-PAN/PEDOT:PSS nanofibers was again observed, as the water droplets deposited onto the fiber mesh were promptly adsorbed by the material. The sulfuric acid-treated PAN nanofibers also displayed a similar hydrophilic profile. While no studies could be found in the literature on the effect of sulfuric acid on the wettability of PAN, these results appear to be in line with those reported for other sulfuric acid-treated hydrophobic materials such as polystyrene [61]. Prior studies have also reported the hydrophilic nature of PEDOT:PSS films after being treated with sulfuric acid [62]. 

Important shifts in the FTIR spectra of the HAT-PAN and HAT-PAN/PEDOT:PSS nanofibers were observed comparatively to their non-treated nanofiber mesh counterparts (Figure 4). Three characteristic PAN-related IR peaks were once again detected in the collected spectra for both HAT-PAN and HAT-PAN/PEDOT:PSS nanofibers: at ca. 1357 cm^−1^, 1453 cm^−1^ and 1623 cm^−1^ [47]. However, two other major IR peaks usually attributed to PAN, and that were previously identified in non-treated nanofibers, were absent (2242 cm^−1^ and 2938 cm^−1^, -C≡N stretching and −CH_2_ stretching, respectively) [47]. This could be explained by the annealing treatment of the nanofibers, which is responsible for significant changes in the original structure of the PAN chains (e.g., nitrile group reaction), including cyclization and dehydrogenation (thermal stabilization) [63]. On the other hand, two new stabilized PAN-related IR peaks could be identified in the FTIR spectra of both HAT-PAN and HAT-PAN/PEDOT:PSS scaffolds, thus confirming the presence of a modified polymer as opposed to pristine PAN: 805 cm^−1^ (–C=C–H– bending vibration, heptazine rings) and 1582 cm^−1^ (C=N, C=C, N–H mixed stretching) [63]. This appears to be in agreement with the published literature, where similar changes in the structure of PAN after annealing are described [40,47,63,64]. 

Four of the five IR peaks assigned to PEDOT:PSS, that were previously detected for the non-treated PAN/PEDOT:PSS scaffolds, were identified on the FTIR spectra of the composite HAT-PAN/PEDOT:PSS fibers (Figure 4). The IR peak at 832 cm^−−1^ is not clearly detected or it overlaps with the 805 cm^−1^ peak attributed to annealed PAN. A reduction in the intensity of the PEDOT:PSS-related IR peak at 1032 cm^−1^, associated with PSS, was registered after HAT treatment. This observation is in line with a lower PSS content in the structure of the post-processed fibers, presumably as PSS is selectively removed upon PEDOT:PSS treatment with sulfuric acid [58,65,66]. The removal of the insulating PSS layer of PEDOT:PSS, accompanied by conformational changes and increased crystallization, facilitates the charge mobility within and between PEDOT domains, thus enabling a significant electroconductivity increase in the CP [67]. Similar results were reported by Laforgue et al. (2016) and Hosseini et al. (2020) with sulfuric acid-doped PEDOT:PSS films [36,65]. Overall, the shifts in the FTIR spectra of the HAT-PAN and HAT-PAN/PEDOT:PSS nanofibers appear to be in line with those observed by our group on the same set of scaffolds with Raman spectroscopy [40].

Four-contact electroconductivity measurements on the HAT-PAN nanofibers revealed an insulating behavior. The HAT-PAN/PEDOT:PSS nanofibers, however, were found to be electroconductive, exhibiting an average electrical conductivity of 8.28 ± 6.70 × 10^−5^ S cm^−1^ (*n* = 3). While the electrical conductivity values obtained were not as high as expected, comparatively with the reported conductivity of sulfuric acid-treated PEDOT:PSS films and wet-spun fibers, they were comparable with the conductivity values (~10^−5^ S cm^−1^) reported in the literature for PEDOT:PSS-based electrospun fibers, mostly manufactured by coating the fibers with PEDOT:PSS solutions of similar concentrations as the one used in this study [39,59,65]. Importantly, the electrical conductivity value obtained for the composite HAT-PAN/PEDOT:PSS nanofibers is similar to the one reported for cortical bone (9.1 × 10^−5^ S cm^−1^) by Balmer et al. (2018) [68]. 

### 2.3. In Vitro Scaffold Mineralization

In order to properly functionalize the scaffolds for BTE applications and assess the effect of PEDOT:PSS on the mineralization process, the HAT-treated nanofibers were incubated in concentrated SBF 2.5× for a 7-day period.

**Figure 5:**
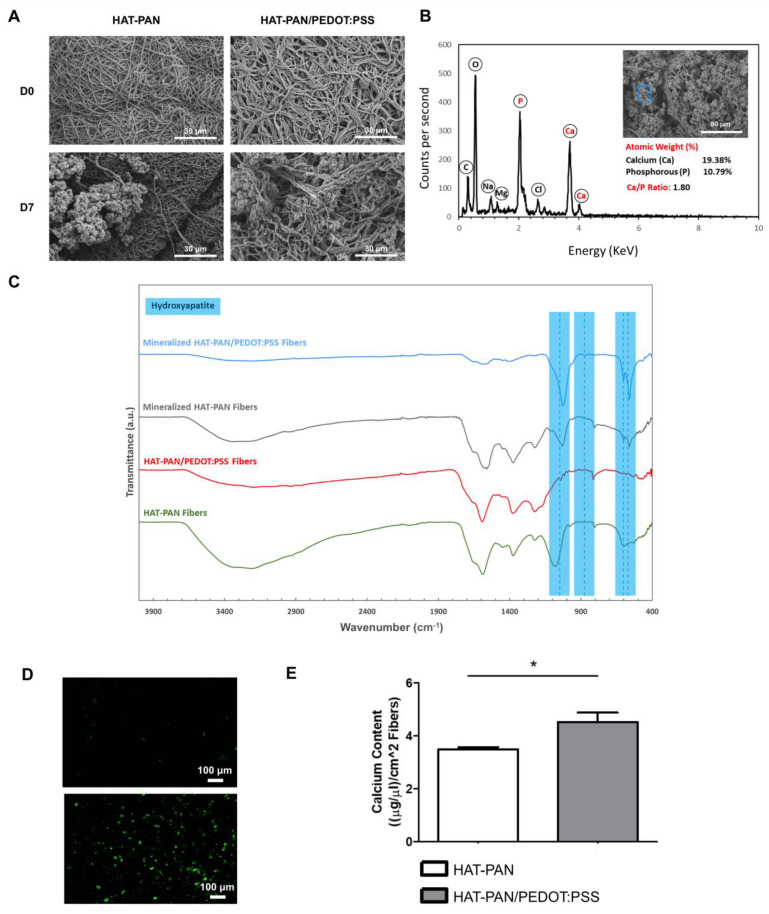
SEM micrographs (**A**) of the HAT-PAN and HAT-PAN/PEDOT:PSS scaffolds at day 0 (D0) and after 7 days (D7) of incubation in SBF 2.5×. EDX analysis (**B**) of the generated composite HAT-PAN/PEDOT:PSS scaffolds after 7 days of mineralization. A SEM image of the spot where EDX analysis was conducted is presented inside the EDX spectrogram (highlighted in blue). The atomic percentage of calcium and phosphorous present in the sample is highlighted, and the respective calcium/phosphorous (Ca/P) ratio is estimated. FTIR analysis (**C**) of the HAT-PAN and HAT-PAN/PEDOT:PSS nanofibers after 0 and 7 days of incubation in SBF 2.5×. Four hydroxyapatite-related IR characteristic peaks are highlighted in blue (570 cm^−1^, 604 cm^−1^, 875 cm^−1^ and 1049 cm^−1^). OsteoImage mineralization staining (**D**) of the HAT-PAN (**top**) and HAT-PAN/PEDOT:PSS (**bottom**) nanofibers after 7 days of mineralization. OsteoImage stains hydroxyapatite deposits green. Calcium content (**E**) of the HAT-PAN and HAT-PAN/PEDOT:PSS nanofibers after being incubated for a 7-day period in SBF 2.5×. Three different samples (n=3) were used in the analysis presented in (**E**); * *p* < 0.05. Scale bar values are depicted in the SEM/fluorescence microscopy images.

As shown in Figure 5, the in vitro mineralization process was successful. After 7 days of incubation in SBF 2.5×, clear formation of large mineral aggregates on the surface of both HAT-treated PAN and HAT-treated PAN/PEDOT:PSS nanofibers was confirmed by SEM imaging (Figure 5A). EDX analysis of the HAT-PAN and HAT-PAN/PEDOT:PSS scaffolds after mineralization confirmed the presence of calcium and phosphorous, the major components of hydroxyapatite (HAp), on the surface of the nanofibrous scaffolds (Figure 5B and Supplementary Material, Figure S2). Sodium- and magnesium-containing deposits, which are also commonly found in the inorganic phase of the bone tissue, were also detected [69,70]. FTIR analysis of the HAT scaffolds at the end of the incubation period further corroborated that mineralization had taken place, with three key IR peaks assigned to the phosphate group present in HAp being identified: 570 cm^−1^ and 604 cm^−1^ (–P–O asymmetric bending vibration) and 1049 cm^−1^ ((PO_4_)^3-^ asymmetric stretching) (Figure 5C) [71,72]. An additional peak assigned to the carbonate group was also detected in the FTIR spectra of both mineralized HAT-PAN and HAT-PAN/PEDOT:PSS nanofibers: 875 cm^−1^ (CO_3_^2-^ asymmetric bending) [73]. The calcium/phosphorus (Ca/P) ratio of the mineral deposits formed on the surface of the HAT-treated scaffolds was estimated using the collected EDX data (Figure 5B and Supplementary Material, Figure S2). The Ca/P ratios that were obtained—1.72 and 1.80 for the HAT-PAN and HAT-PAN/PEDOT:PSS nanofibers, respectively—were within the range of values reported in the literature for human bone tissue (1.3–2.2) [74]. Overall, these results appear to suggest that the obtained HAT scaffolds would be capable of supporting the mineralization process when placed in an in vivo setting, fostering the formation of HAp-like constructs with biomimetic composition similar to those found in the native bone ECM [75].

In order to assess the impact of PEDOT:PSS on the mineralization process, both mineralized HAT-PAN and HAT-PAN/PEDOT:PSS nanofibers were stained with a HAp-specific OsteoImage staining reagent. As shown in Figure 5D, both experimental conditions stained positive for HAp, in accordance with the results obtained from SEM, EDX and FTIR analyses. However, a more efficient mineralization seems to be attained for the HAT-PAN/PEDOT:PSS scaffolds, comparatively with the control HAT-PAN nanofibers, as a larger number of mineral deposits were visible in the surface of the former. This was confirmed by a calcium quantification assay, where a higher calcium content (statistically significant) was obtained for the electroconductive HAT-PAN/PEDOT:PSS nanofibers compared with the control HAT-treated scaffolds (Figure 5E), suggesting that the addition of PEDOT:PSS might have had a positive effect towards the formation of a HAp-like mineral coating on the surface of the composite nanofibers. This could be potentially explained by the CP decreasing the surface energy required for inducing the heterogenous nucleation of apatite from the SBF [76]. Multiple studies have described the successful mineralization of PEDOT:PSS-containing scaffolds using SBF [77,78,79]. Recently, Hassan et al. (2017) reported an enhanced mineralization for PEDOT:PSS-coated polylactic acid (PLA)/polyhydroxybutyrate (PHB)/HAp (PLA/PHB/HAp) nanofibers comparatively with their non-coated electrospun counterpart [79]. A similar phenomenon has also been reported using other CPs, such as polyaniline and polypyrrole [80,81].

### 2.4. Osteoblast-like MG-63 Cell Viability and Proliferation on Mineralized PAN/PEDOT:PSS Nanofibers

In order to evaluate the effect of mineralization of HAT-PAN and HAT-PAN/PEDOT:PSS electrospun nanofibers on cell viability and proliferation, osteoblast-like MG-63 cells were seeded on the surface of the fibrous scaffolds and cultured for a 10-day period. Cultures on non-mineralized HAT-PAN and HAT-PAN/PEDOT:PSS electrospun nanofibers were used as controls.

**Figure 6:**
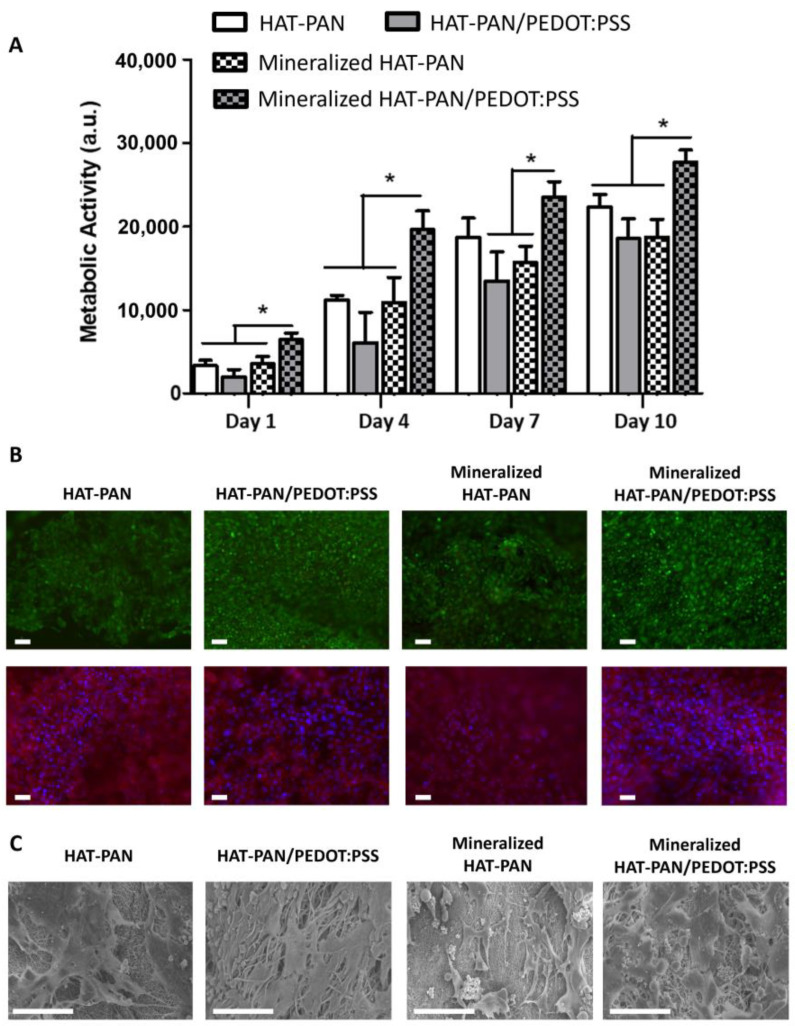
Cell metabolic activity assay (**A**), Live/Dead (**top**) and DAPI/Phalloidin (**bottom**) stainings (**B**) and SEM micrographs (**C**) of osteoblast-like MG-63 cells cultured on mineralized/non-mineralized HAT-PAN and HAT-PAN/PEDOT:PSS nanofibers for 10 days. DAPI stains cell nuclei blue while Phalloidin stains actin-rich cytoskeleton red. Calcein stains viable cells green while ethidium homodimer-1 stains dead cells red. Scale bar: 100 μm (Live/Dead), 50 μm (DAPI/Phalloidin) and 80 μm (SEM). Four different independent samples (n=4) were used in the cell metabolic activity assay analysis; * *p* < 0.05.

Cell proliferation was monitored at days 1, 4, 7 and 10 of cell culture using an AlamarBlue^TM^ assay. As shown in Figure 6A, cell growth was observed over time for all experimental groups. Regarding the non-mineralized scaffolds, slightly augmented values of metabolic activity (not statistically significant) were obtained for the HAT-PAN scaffolds compared with the composite HAT-PAN/PEDOT:PSS nanofibers (Figure 6A). However, for the mineralized scaffolds, a statistically significant higher metabolic activity was registered for the PEDOT:PSS-containing scaffolds (Figure 6A) in comparison to mineralized HAT-PAN scaffolds. In fact, while comparable values of metabolic activity were obtained for the mineralized HAT-PAN scaffolds relative to their non-functionalized counterpart, the mineralized HAT-PAN/PEDOT:PSS nanofibers promoted significantly higher cell metabolic activities in relation to all other experimental conditions. The obtained results appear to suggest that the sole addition of PEDOT:PSS to the fiber composition did not have a particularly significant impact on the bioactivity of the PAN-based scaffolds. Such observation is in accordance with a previous study conducted in our group, in which hBM-MSCs cultured for 7 days on PEDOT:PSS-coated polybenzimidazole (PBI) nanofibers showed similar cell metabolic activities to the ones cultured on non-coated PBI scaffolds [82]. Nevertheless, by enabling a more comprehensive mineralization of the scaffolds in SBF, the presence of the CP might also explain the more expressive cell growth observed for the mineralized HAT-PAN/PEDOT:PSS nanofibers, which was not observed for the mineralized HAT-PAN scaffolds likely due to a less effective mineralization process. Several studies have reported the improved bioactivity of HAp-coated scaffolds. Jaiswal et al. (2013) observed enhanced bioactivity of HAp-coated polycaprolactone (PCL)/gelatin nanofibers cultured with human osteosarcoma cells [83]. In a different study, Ebrahimi et al. (2022) registered an increased proliferation rate of adipose tissue-derived MSCs on HAp-coated 3D-printed PCL scaffolds [84].

Live/Dead and DAPI/Phalloidin stainings of the osteoblast-like MG-63 cells were performed after 10 days of cell culture to evaluate cell viability as well as their morphology and distribution on the different scaffolds (Figure 6B). A high density of cells was observed on the surface of the different electrospun scaffolds, confirming the successful adhesion and proliferation of the osteoblast-like cells on the surface of the material. Most cells were also found to be viable at the end of the cell culture period, evidencing the biocompatibility of the scaffolds. Higher percentages of live cells were obtained for the PEDOT:PSS-containing scaffolds (86.55 ± 9.35% and 93.67 ± 1.75% for the non-mineralized and mineralized nanofibers, respectively) comparatively to the control PAN fibers (76.09 ± 11.02% and 90.82 ± 3.15% for the non-mineralized and mineralized nanofibers, respectively) (Supplementary Material, Figure S3). These results are in line with what was observed in prior studies, which were also able to validate the biocompatibility of both annealed PAN and PEDOT:PSS [85,86,87,88]. Through SEM imaging, it was possible to observe that the osteoblast-like cells were spread across the entire surface of the different nanofibers, apparently exhibiting random orientations (Figure 6C).

### 2.5. hBM-MSCs Osteogenic Differentiation on Mineralized PAN/PEDOT:PSS Nanofibers

In order to evaluate the effects of adding PEDOT:PSS and mineralizing the electrospun nanofibers on their ability to support MSCs osteogenic differentiation, hBM-MSCs were seeded on the mineralized and non-mineralized HAT-PAN and HAT-PAN/PEDOT:PSS scaffolds and cultured for a 21-day period under an osteogenic induction medium.

**Figure 7:**
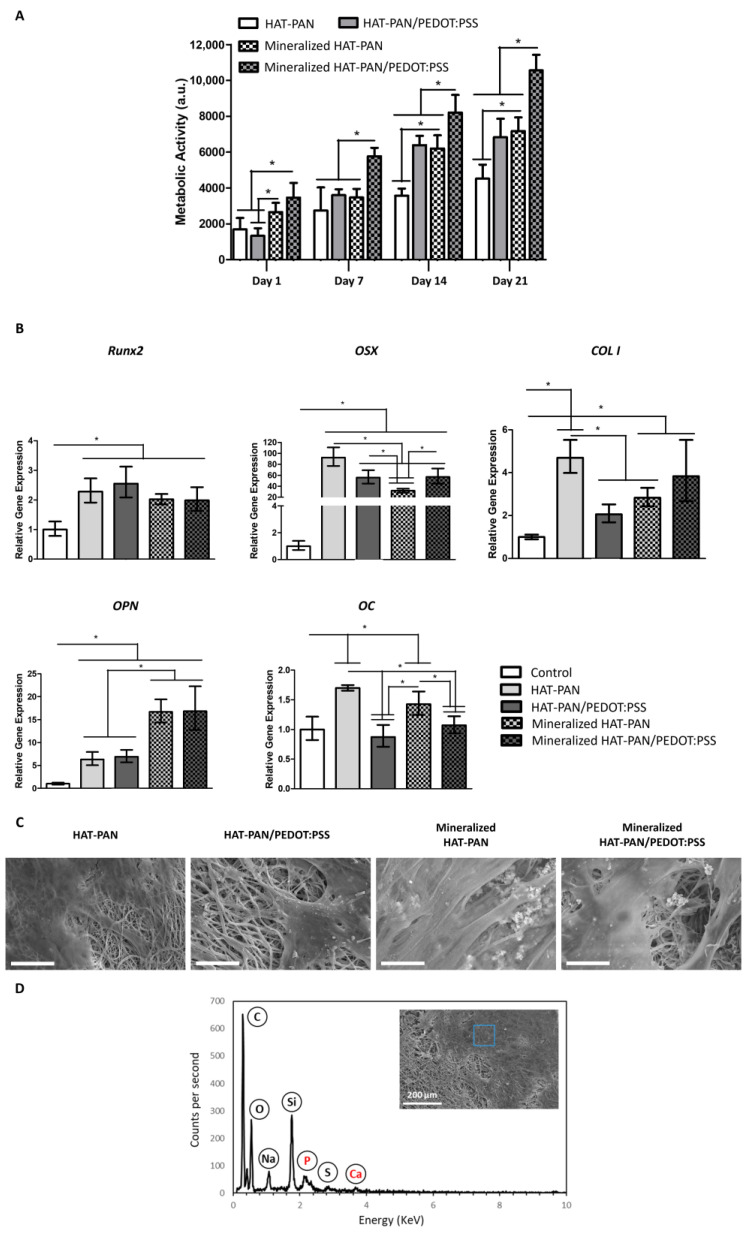
Cell metabolic activity assay (**A**), qRT-PCR analysis (**B**) and SEM micrographs (**C**) of hBM-MSCs cultured on HAT-PAN and HAT-PAN/PEDOT:PSS nanofibers with and without mineral coating after 21 days of osteogenic differentiation. In the qRT-PCR analysis, the expressions of the target genes *Runx2*, *OSX*, *COL I*, *OPN* and *OC* were normalized to *GADPH* and calculated as fold-change relative to the baseline expression of the control sample (hBM-MSCs before scaffold seeding at day 0). EDX analysis (**D**) of cell-seeded HAT-PAN/PEDOT:PSS scaffolds after 21 days of osteogenic differentiation. A SEM image of the spot where EDX analysis was conducted is presented inside the EDX spectrogram (highlighted in blue). Three different samples (*n* = 3) were used in the RT-qPCR analysis. Scale bar: 30 μm; * *p* < 0.05.

hBM-MSC metabolic activity was monitored using the AlamarBlue^TM^ assay at days 1, 7, 14 and 21 of cell culture. As shown in Figure 7A, cell growth was observed for all experimental groups, with a stepper increase in cell metabolic activity observed between days 7 and 14. Higher values of cell’s metabolic activity were obtained for the mineralized HAT-PAN/PEDOT:PSS nanofibers compared with the mineralized HAT-PAN scaffolds, with statistically significant differences between both conditions being identified at days 7, 14 and 21 of cell culture. A similar trend was also observed for the non-mineralized nanofibers, with more significant increases in metabolic activity (statistically significant at days 14 and 21) being registered for the PEDOT:PSS-containing scaffolds compared with the control HAT-PAN scaffolds. Comparable values of metabolic activity were obtained for the non-mineralized HAT-PAN/PEDOT:PSS nanofibers and mineralized HAT-PAN scaffolds (Figure 7A). Unlike what was previously observed with the MG-63 osteoblast-like cells, these results appear to suggest that the addition of PEDOT:PSS had a non-negligible positive contribution to the bioactivity of the PAN scaffolds. This difference in cell response could be explained by the fact that different cell types interact differently with the same materials [89]. The shorter culture period for the MG-63 osteoblast-like cells experiment (10 days) may also explain the differences observed, since the increased metabolic activity registered for the MSC-seeded HAT-PAN/PEDOT:PSS nanofibers was only detected after 14 days of culture. A similar trend (concerning MSC growth) resulting from the PEDOT:PSS addition to chitosan electrospun scaffolds was reported by Abedi et al. (2019) using rat BM-MSCs [39]. Significantly higher values of metabolic activity were registered for the mineralized HAT-PAN/PEDOT:PSS scaffolds comparatively with all other experimental conditions, which is likely the result of a higher degree of surface mineralization. The results observed for hBM-MSC metabolic activity corroborate the synergy between the use of PEDOT:PSS and mineralization to promote cell adhesion and proliferation, in agreement with the previous experiments performed with MG-63 osteoblast-like cells. 

qRT-PCR analysis of samples collected after 21 days of osteogenic differentiation was performed to evaluate the expression of key bone-specific markers commonly expressed at different stages of the osteogenic differentiation process, namely *Runx2*, osterix (*OSX*), type I collagen (*COL I*), osteopontin (*OPN*) and osteocalcin (*OC*) (Figure 7B). Up-regulation of *Runx2*, *OSX*, *COL I* and *OPN* gene expression was observed for all tested scaffolds compared with the control condition (hBM-MSCs at day 0). *Runx2* was found to be similarly expressed among the different conditions. *OSX* expression appeared to be impacted in different ways by the mineralization of the nanofibers: while the HAT-PAN/PEDOT:PSS nanofibers exhibited similar levels of *OSX* expression after being mineralized, the control HAT-PAN scaffolds registered a noticeable drop (statistically significant) in *OSX* gene expression with scaffold mineralization. *OPN* expression was found to be significantly up-regulated (statistically significant) for the mineralized scaffolds compared to their non-functionalized counterparts. Similar *OPN* gene expression was registered for both mineralized HAT-PAN and mineralized HAT-PAN/PEDOT:PSS nanofibers. *COL I* gene expression appeared to increase with the mineralization of the HAT-PAN/PEDOT:PSS nanofibers, while the opposite trend was observed for the control HAT-PAN scaffolds. Both mineralized and non-mineralized HAT-PAN nanofibers displayed slightly augmented expressions of *OC* relative to the control, while such an increase was not observed for both HAT-PAN/PEDOT:PSS nanofibers. Overall, the up-regulated expression of a more mature stage osteogenic marker, such as *OPN*, and the slightly reduced expression of early osteogenic markers, like *Runx2*, suggest that the mineralized scaffolds are likely populated by more mature pre-osteoblasts after 21 days of differentiation, therefore, supporting their improved osteogenic potential. Several other studies have also registered an up-regulated expression of key osteogenic markers as a result of the functionalization of BTE scaffolds with mineral components (e.g., mineral coating) [90,91]. Recently, Wu et al. (2020) reported an increase in *COL I* and *OPN* gene expression by mouse osteoblastic MC3T3-E1 cells seeded on SBF-mineralized hydroxyethyl cellulose/soy protein isolate scaffolds [92]. In a different study, Wang et al. (2019) observed an up-regulated expression of *COL I*, *OPN* and *Runx2* by rat BM-MSCs seeded on electrospun poly(lactic-co-glycolic acid) (PLGA)/PCL nanofibers filled with octacalcium phosphate particles [93]. The differential effect of mineralization of the HAT-PAN and HAT-PAN/PEDOT:PSS nanofibers on *OSX* and *COL I* gene expression appears to show a positive contribution of the CP for the MSC’s osteogenic differentiation process. This is in line with results reported by Guex et al. (2017), which were also able to corroborate the osteogenic potential of highly porous PEDOT:PSS scaffolds [94]. 

Immunofluorescence staining of the HAT-mineralized/non-mineralized PAN/PEDOT:PSS and PAN scaffolds was performed after 21 days of culture to confirm the presence of COL I and OPN within the obtained tissue constructs. As demonstrated in Figure S4 (Supplementary Material), all experimental conditions were stained positive for both COL I and OPN, with no meaningful differences in marker intensity being observed between them. 

SEM and EDX analyses of the scaffolds were performed at the end of the differentiation period in order to assess cell morphology and distribution, as well as the composition of the cell-secreted ECM deposited on the surface of the nanofibers. Through SEM imaging, a high density of differentiated cells could be observed on the surface of the different scaffolds (Figure 7C). Cell-derived mineralization on the surface of the nanofibers was observed for all experimental conditions with the detection of calcium and phosphorous deposits via EDX analysis (Supplementary Material, Figure S5). In Figure 7D, an example of an EDX spectrum collected from the non-mineralized HAT-PAN/PEDOT:PSS nanofibers is presented.

Overall, the obtained results seem to corroborate the osteoinductive properties of the developed mineralized HAT-PAN/PEDOT:PSS nanofibers, emphasizing their interesting potential for future BTE applications. Future studies with these scaffolds should include the systematic ES of the nanofibers during cell culture to further assess the possible combined role of PEDOT:PSS and ES on the osteogenic differentiation of MSCs. Despite the high biocompatibility observed for the PAN/PEDOT:PSS scaffolds, the degradation profile of the nanofibers should also be investigated under in vivo-like or accelerated degradation conditions to fully assess the potential release of toxic chemicals.

## 3. Materials and Methods

### 3.1. Materials

PEDOT:PSS (Clevios™ PH1000) was acquired from Heraeus (Hanau, Germany). PAN (MW 200.000 Da) was purchased from Polysciences (Warrington, PA, USA). Dimethylformamide (DMF) and dimethyl sulfoxide (DMSO) were obtained from Carlo Erba Reagents (Milan, Italy) and Sigma Aldrich (St. Louis, MI, USA), respectively. Concentrated sulfuric acid (95–97%) was acquired from Honeywell Fluka (Charlotte, NC, USA).

### 3.2. Preparation of Electrospinning Solutions

In order to produce the electroconductive fibers, first, PEDOT:PSS solutions were prepared by adapting the protocol described by Lu et al. (2019) [52]. Briefly, DMSO was added to PEDOT:PSS at a fixed concentration of 10% (*v/v*). The resulting mixture was then dried in a mechanical convection oven (HERATherm Oven; Thermo Scientific, Waltham, MA, USA) at 60 °C overnight. The resulting PEDOT:PSS pellets were dry-annealed in the oven at 130 °C in three consecutive 30 min cycles. The annealed PEDOT:PSS pellets were then ground into a powder, which was, in turn, dispersed in DMF:DMSO (9:1) at a concentration of 3 wt%. The resulting solutions were then agitated for at least 7 days until a homogenous mixture was obtained. Before electrospinning, PAN was added at a fixed concentration of 10 wt%, and the mixture was heated on a hot plate (RCT Classic; IKA, Staufen, Germany) for approximately 20 min at 85 °C. PAN solutions were prepared by simply mixing PAN in DMF:DMSO (9:1); after which, the resulting solutions were heated on a hot plate at 85 °C for approximately 20 min. A summary of the process used to develop the PAN/PEDOT:PSS electrospinning casting solutions is presented in Scheme S1 (Supplementary Material).

### 3.3. Electrospinning Setup and Parameters

PAN/PEDOT:PSS nanofibers were produced using a spinneret system (MECC, Ogori, Fukuoka, Japan) and by applying a voltage of 20 kV to a 21G stainless steel needle at a 25 cm distance from a grounded static copper collector covered with aluminum foil. A controlled flow rate of 1 mL/h was used to eject the polymeric solution. The electrospinning apparatus also included a syringe pump (Model NE-1000, New Era Pump Systems, New Farmingdale, NY, USA), a high voltage power source (Series EL, Model ES/EL40P01, XP Glassman High Voltage Inc., High Bridge, NJ, USA) and polytetrafluoroethylene (PTFE) tubing (NICHIAS Corporation, Shanghai, China). The temperature and relative humidity were monitored and controlled during electrospinning, and the parameters varied between 20 and 23 °C and 35 and 55%, respectively.

### 3.4. Fiber Post-Processing: Heat and Sulfuric Acid Treatment (HAT)

The PAN/PEDOT:PSS nanofibers were dipped in sulfuric acid in order to improve their electrical conductivity (Supplementary Material, Scheme S1) [40]. First, the fibers were annealed for a 24 h period in an oven at 210 °C to stabilize PAN (cyclization). After cooling, the fibers were collected from the aluminum foil and were positioned in glass plates. Next, the fibers were incubated with sulfuric acid for 24 h. During this period, the sulfuric acid solution was changed three times. Afterwards, the acid was removed from the glass plates, which were then placed in an oven at 130 °C for 30 min. After cooling, several washes with distilled water were performed to completely remove the acid and residues. Finally, the fibers were dried overnight at 37 °C. The same procedure was applied to the PAN fibers. 

### 3.5. Characterization of PAN/PEDOT:PSS Nanofibers

#### 3.5.1. Scanning Electron Microscopy (SEM)

SEM analysis of the structure of the electroconductive PAN/PEDOT:PSS fibers was performed using a field emission gun SEM (FEG-SEM) (Model JSM-7001F; JEOL, Tokyo, Japan). The samples were first mounted on a holder using carbon tape and were coated in a 30 nm gold/palladium (60:40) layer (Model E5100 Sputter Coater; Polaron/Quorum Technologies, Lewes, UK). The samples were imaged at several magnifications using an average accelerating voltage of 15 kV. The average fiber diameter of the scaffolds was computed using ImageJ software version 1.53j (NIH, Bethesda, MD, USA). The average diameter of 100 individual fibers (n=100) was estimated for each condition from at least five distinct SEM images.

#### 3.5.2. Elemental Composition Analysis

Energy Dispersive X-ray (EDX) analysis (INCA Microanalysis Suite software, Oxford 250/HKL INCA Energy) of the generated PAN/PEDOT:PSS fibers was conducted to establish the presence of the CP as well as corroborate the formation of a bone-like mineral coating on the surface of the scaffolds previously immersed in SBF (described in Section 3.6). EDX analysis was also performed to evaluate MSC-derived mineralization on the surface of the fibrous scaffolds after three weeks of osteogenic differentiation (Section 3.7.2). This analysis was performed with an average acceleration voltage of 10 kV and a spot size of 30 µm.

#### 3.5.3. Contact Angle Measurements

The contact angle of the electroconductive nanofibers was measured using the sessile drop method with distilled water (DSA25B Goniometer; Kruss, Hamburg, Germany). The left and right contact angles of the droplets with the surface of the scaffolds were determined, and the average value was computed. The acquisition rate used was 2 measurements per second. The contact angle was measured in 7 independent fiber samples (n=7) for each individual experimental group, and snapshot pictures were taken at the end of each measurement. 

#### 3.5.4. Attenuated Total Reflection Fourier-Transform Infrared (ATR-FTIR) Spectroscopy

ATR-FTIR spectra were collected using a Spectrum Two FT-IR Spectrometer from PerkinElmer (Waltham, MA, USA) equipped with an attenuated total reflection UATR Two accessory. This analysis was conducted to identify important functional groups commonly found in both PAN and PEDOT:PSS to further corroborate the presence of these materials within the fiber composition as well as monitor the impact of the HAT treatment on the chemical structure of the polymers. Transmittance spectra were obtained at room temperature over the spectral region from 400 cm^−1^ to 4000 cm^−1^, with a resolution of 4 cm^−1^ and 8 scans of data accumulation, followed by an automatic baseline correction using the manufcaturer’s acquisition software (Spectrum 10^™^ software). All ATR-FTIR spectra were normalized using the maximum and minimum transmittance of each spectrum.

#### 3.5.5. Four-Probe Electroconductivity Measurements

Four 50 nm thick and equidistant gold stripes were deposited on the surface of the electrospun nanofiber mats by physical vapor deposition (PVD) using a thermal evaporator (Model E306A; Edwards, Irvine, CA, USA). The electroconductivity was measured with the four-contact method using a current source Keithley 2400 DC power source (Keithley Instruments, Cleveland, OH, USA) and a multimeter (Model 34401A; Agilent Technologies, Santa Clara, CA, USA). The probes were put in direct contact with the gold stripes. The resistance (R) of the samples was calculated according to Ohm’s Law (R = ΔV/I) by correlating the differential potential measured between the inner gold contacts (ΔV) with the electrical current being applied on the outer contacts (I). Experimental data were collected and processed using LabVIEW 7.1 software. The obtained ΔV and I values were used to plot I/V curves, and their slopes were calculated (1/R). Conductivity (σ) was computed by using its relationship with R, at constant temperature (Equation (1)):

(1) $$\sigma =\frac{1}{R}\left(\frac{L}{A}\right)\;\left[S/m\right]\;$$

where L corresponds to the distance between the two inner gold contacts and A corresponds to the sample’s cross-section area. The thickness of the fiber mats was measured with a caliper. Measurements were performed in triplicates (n=3) and averaged. 

### 3.6. In Vitro Mineralization

In order to mimic the inorganic phase of the bone ECM, the produced PAN and PAN/PEDOT:PSS fibers were incubated in concentrated SBF (SBF 2.5×) for a 7-day period to promote the formation of a mineral coating (Supplementary Material, Scheme S1). The SBF solution was prepared following the protocol described by Kokubo et al. (2006) [75]. Briefly, the fibers were first washed in ethanol 20% (*v/v*) and distilled water to remove impurities. Afterwards, the scaffolds were immersed in SBF 2.5× and placed in an incubator at 37 °C. The medium was fully replaced every two days. 

To confirm the successful mineralization of the scaffolds, an OsteoImage™ Mineralization Assay kit (Lonza, Basel, Switzerland) was used to stain the mineral deposits following the manufacturer’s guidelines. Briefly, the samples were first transferred to 24-well plates; after which, they were washed with a wash buffer provided in the kit. The scaffolds were then incubated for 30 min with the staining reagent in the dark at room temperature. The samples were washed with phosphate-buffered saline solution (PBS, Gibco; ThermoFisher, Waltham, MA, USA), and the fluorescence staining was imaged using an inverted fluorescence microscope (LEICA DMI3000B, Leica Microsystems, Wülfrath, Germany).

A calcium colorimetric assay kit (Sigma Aldrich) was used to compare the degree of mineralization of PAN and PAN/PEDOT:PSS nanofibers. The mineralized scaffolds were first incubated in a 1M HCl solution overnight under constant agitation. The supernatant was then collected and used for determining calcium concentration, following the manufacturer’s guidelines. Essentially, several dilutions of a Calcium Standard Solution (500 mM) provided in the kit were pipetted into a 96-well plate. Four samples of each experimental condition (n=4) were also added to the 96-well plate. A Chromogenic Reagent and a Calcium Assay buffer (also provided in the kit) were sequentially added to each well, and the solutions were mixed gently by pipetting. The samples were incubated in the dark for 10 min at room temperature. The absorbance was measured on a microplate reader (Infinite 200 Pro; Tecan, Männedorf, Switzerland) at 575 nm (duplicate measurements per sample). A calibration curve was generated from the absorbance measurements collected for the different Calcium Standard Solution concentrations. The obtained calibration curve was used to estimate the concentration of calcium present in each sample. The values were normalized to the surface area of each individual scaffold, which was measured prior to mineralization. The supernatants of non-mineralized PAN and PAN/PEDOT:PSS fibers were used in this assay as blank controls.

### 3.7. In Vitro Cell Culture Studies

#### 3.7.1. Cell Culture

Human MG-63 osteoblast-like cells were acquired from ATCC (CRL-1427™). hBM-MSCs were isolated according to protocols previously established at the Institute for Bioengineering and Biosciences (iBB) at Instituto Superior Técnico (IST) [95,96]. Bone marrow aspirates (male, 46 years) were obtained from Centro Clínico da GNR (Lisboa, Portugal), under collaboration agreements with iBB-IST. All human samples were obtained from healthy donors after written informed consent according to Directive 2004/23/EC of the European Parliament and of the Council of 31 March 2004, on setting standards of quality and safety for the donation, procurement, testing, processing, preservation, storage and distribution of human tissues and cells (Portuguese Law 22/2007, 29 June), with the approval of the Ethics Committee of the respective clinical institution. Isolated cells were kept frozen in liquid/vapor nitrogen tanks until further use. Isolated hBM-MSCs and osteoblasts were cultured using low-glucose Dulbecco’s Modified Eagle Medium (DMEM, Gibco; ThermoFisher, Waltham, MA, USA), supplemented with 10% (*v/v*) fetal bovine serum (FBS; ThermoFisher—MSC qualified for hBM-MSCs) and 1% (*v/v*) antibiotic-antimycotic (Anti-Anti; ThermoFisher). The cells were kept in an incubator at 37 °C and 5% CO_2_ in a humidified atmosphere, and the medium was replaced every 3–4 days. All the experiments were conducted using cells between passages P4 and P6. 

#### 3.7.2. Cell Seeding, Culture and Osteogenic Differentiation

Prior to cell culture studies, the mineralized and non-mineralized PAN and PAN/PEDOT:PSS nanofibers were glued on glass coverslips (10 mm in diameter; VWR) using FDA-approved adhesive silicone glue (Silastic Medical Adhesive Silicone, Type A; Dow Corning). The nanofibrous scaffolds were sterilized by UV light exposure overnight, after which, they were transferred to ultra-low cell attachment 24-well plates (Corning, Somerville, MA, USA). The scaffolds were then washed three times with a 1% (*v/v*) anti-anti solution (prepared in PBS), followed by incubation with cell culture medium for 1 h. After being harvested from their respective T-Flasks, MG-63 osteoblasts and hBM-MSCs were seeded on the fibrous scaffolds at a density of 120,000 and 150,000 cells per scaffold, respectively. The cell-seeded scaffolds were incubated for 2 h without culture medium to promote initial cell attachment. A standard cell culture growth medium (specified in Section 3.7.1) and an osteogenic medium, comprising DMEM supplemented with 10% MSC-qualified FBS, 10 mM of β-glycerolphosphate (Sigma Aldrich), 10 nM of dexamethasone (Sigma Aldrich), 50 µg mL^−1^ of ascorbic acid (Sigma Aldrich) and 1% anti-anti, were added to the osteoblast and MSC-seeded scaffolds, respectively. Cell culture was conducted during a 10-day and 21-day period for the MG-63 osteoblast-like cells and MSC-seeded scaffolds, respectively, at 37 °C and 5% CO_2_ in a humidified atmosphere. Cell culture medium was fully renewed every 3–4 days. 

#### 3.7.3. Proliferation Assay

An AlamarBlue^TM^ assay (AlamarBlue^TM^ Cell Viability Reagent; ThermoFisher) was conducted to monitor the metabolic activity of the MG-63 osteoblasts and hBM-MSCs on the mineralized nanofibers and respective controls. This analysis was performed on days 1, 4, 7 and 10, and 1, 7, 14 and 21 of cell culture for the MG-63 osteoblast-like cells and hB-MSC-seeded scaffolds, respectively. The AlamarBlue^TM^ assay was conducted following the manufacturer’s guidelines. Briefly, a 10% (*v/v*) AlamarBlue^TM^ solution in cell culture medium was added to the cell-seeded scaffolds and respective controls and incubated for 4 h and 3 h, respectively, for the MG-63 osteoblast-like cells and hBM-MSC-seeded scaffolds. Fluorescence intensity was measured in a microplate reader (Infinite 200 Pro; Tecan) at an excitation/emission wavelength of 560/590 nm. Fluorescence intensity was analyzed for five independent scaffolds for each experimental group (n=5), and the values of each well were acquired in triplicates. Acellular electrospun scaffolds were used as blank controls.

#### 3.7.4. Cell Morphology and Viability Assessment

The morphology of the MG-63 osteoblast-like cells and differentiating hBM-MSCs on the different scaffold conditions was analyzed through SEM imaging at the end of the respective cell culture experiments. After being fixed in 4% paraformaldehyde (PFA; Sigma Aldrich) for 20 min, the samples were immersed in aqueous ethanol solutions with gradually increasing concentrations (20%, 40%, 60%, 80%, 90% and 100% (*v/v*)) in 30 min intervals. The scaffolds were then immersed in hexamethyldisilazane (HMDS; Sigma Aldrich) for 1 h and left to dry overnight in a fume hood. The dried cell-seeded electrospun fibers were coated with a gold/palladium layer prior to being imaged, as previously described in Section 3.5.1.

Live/Dead staining of the MG-63 osteoblast-seeded scaffolds was also performed to corroborate the biocompatibility of the composite scaffolds and assess cell viability after 10 days of culture. The cell-seeded scaffolds were first washed with PBS; after which, they were incubated in the dark with an ethidium bromide (2 µM mL^−1^) (Sigma Aldrich) and calcein (4 µM) (Sigma Aldrich) solution (prepared in PBS) for 1 h. Fluorescence images were obtained using a LEICA DMI3000B inverted fluorescence microscope. The live cell percentage was estimated by counting the number of live cells, in three different regions of the multiple scaffolds (n=3), which was subdivided by the total number of cells (live and dead). This metric was computed using ImageJ software. 

In order to further investigate the morphology and distribution of the MG-63 osteoblast-like cells on the different types of scaffolds, the cell-seeded samples were also stained with 4,6-diamidino-2-phenylindole dihydrochloride (DAPI) and Phalloidin after 10 days of cell culture. After being washed in PBS and fixed in 4% PFA solution for 20 min, the cells were permeabilized in a 0.1% Triton X-100 solution (Sigma Aldrich) in PBS for 10 min. Afterwards, the samples were incubated in the dark with Phalloidin-TRITC (2 µg mL^−1^ in PBS) (Sigma Aldrich) for 45 min. The cell-seeded scaffolds were then washed with PBS and counterstained with DAPI (1.5 µg mL^−1^ in PBS) (Sigma Aldrich) for 5 min. Finally, the samples were washed with PBS, and the fluorescence staining was imaged using an inverted fluorescence microscope (LEICA DMI3000B, Leica Microsystems).

#### 3.7.5. RNA Isolation and Quantitative Real-Time PCR (qRT-PCR) Analysis

Total RNA extraction was performed using the RNeasy Mini Kit (QIAGEN, Hilden, Germany) on the different experimental groups of MSC-seeded nanofibrous scaffolds after 21 days of osteogenic differentiation. The samples were first incubated in lysis buffer (RLT buffer) for 1 h under agitation. Afterwards, total RNA was isolated and purified following the manufacturer’s guidelines. RNA concentration was quantified using a NanoVue Plus spectrophotometer (GE Healthcare, Chicago, IL, USA). cDNA was synthesized from the purified RNA using the High-Capacity cDNA Reverse Transcription Kit (Applied Biosystems, Waltham, MA, USA) according to the manufacturer’s protocol. Briefly, previously prepared reaction mixtures were incubated in a T100™ thermal cycler (Bio-Rad, Hercules, CA, USA) for 5 min at 25 °C, 20 min at 46 °C and 1 min at 95 °C and then were maintained at 4 °C until use. Real-time quantitative reverse transcription-polymerase chain reaction (qRT-PCR) analysis was performed using NZYSpeedy qPCR Green Master Mix (2×), ROX plus (NZYTech, Lisbon, Portugal) and StepOnePlus real-time PCR system (Applied Biosystems). All reactions were carried out at 95 °C for 10 min, followed by 40 cycles of 95 °C for 15 s and 60 °C for 1 min. All samples were analyzed in triplicates (n=3). The CT values obtained were normalized against the expression of the housekeeping gene glyceraldehyde-3-phosphate (*GADPH*), and their analysis was performed using the 2^−ΔΔCt^ method. Gene expression results were determined as a fold-change relative to the baseline expression of the target genes in the control sample (hBM-MSCs before scaffold seeding). The primer sequences used in the qRT-PCR analysis are presented in Table 1. 

#### 3.7.6. Immunofluorescence Analysis

The production of type I collagen and osteopontin, two relevant bone ECM-specific proteins, by the hBM-MSCs seeded on the different scaffolds was evaluated after 21 days of osteogenic differentiation through immunofluorescence imaging. PFA-fixed MSC-seeded scaffolds were first washed in PBS and 1% BSA (Sigma Aldrich); after which, they were immersed in a 1% BSA, 10% FBS and 0.3% Triton X-100 permeabilization/blocking solution for 45 min at room temperature. The samples were then incubated overnight with the primary antibodies for osteopontin (ab8448; abcam, Cambridge, UK) and type I collagen (MA1-26771, ThermoFisher) (1:150 in 1% BSA, 10% FBS and 0.3% Triton X-100 solution) at 4 °C. Afterwards, the nanofibrous scaffolds were incubated for 1 h in solutions containing the secondary antibodies (prepared in 1% BSA at a dilution factor of 1:150)—goat anti-rabbit AlexaFluor™ 448 for osteopontin and goat anti-mouse AlexaFluor™ 546 for type I collagen—at room temperature protected from light. After being washed in PBS, the cell-seeded scaffolds were imaged using a fluorescence microscope (LEICA DMI3000B, Leica Microsystems).

### 3.8. Statistical Analysis

Results are presented as mean values ± standard deviation (SD). All the experiments were conducted using three independent samples (n=3), unless specified otherwise. Statistical analysis of the data was performed using one-way ANOVA, followed by Tukey post hoc test using GraphPad Prism 5 software. Data were considered statistically significant when the p-values obtained were lower than 0.05 (95% confidence intervals, * *p* < 0.05).

## 4. Conclusions

In summary, PAN/PEDOT:PSS electrospun nanofibers were successfully produced and characterized. The fabricated scaffolds were also successfully treated with sulfuric acid, being able to both preserve their fibrous structure and achieve a meaningful boost in their electroconductive features. The PAN/PEDOT:PSS scaffolds exhibited bone-like electroconductivity and were capable of emulating the native fibrous architecture of the bone tissue ECM, including its nanoscale dimensions. Our results showed that the presence of PEDOT:PSS in fiber composition facilitated the in vitro mineralization of the scaffolds in SBF, therefore, demonstrating their enhanced bioactivity. Higher levels of MG-63 osteoblast-like cells and hBM-MSCs proliferation were obtained for the mineralized HAT-PAN/PEDOT:PSS nanofibers, further highlighting their biocompatibility and improved bioactive cues. An improved osteogenic performance was also obtained for the mineralized scaffolds, with an up-regulated expression of late osteogenic markers (e.g., *OPN*) by differentiating hBM-MSCs being observed. Overall, this study presents, for the first time, the development of a mineralized electroconductive PEDOT:PSS nanofibrous scaffold with osteoinductive properties, highlighting their potential for innovative ES-based treatments for bone defect repair.

## Figures and Tables

**Table 1:** Primer sequences used in the qRT-PCR analysis.

Gene	Forward Primer Sequence	Reverse Primer Sequence
*GADPH*	5′-GGTCACCAGGGCTGCTTTTA-3′	5′-CCTGGAAGATGGTGATGGGA-3′
*OSX*	5′-CTGGACATGACACACCCCTAT-3′	5′-GCTGGATTAAGGGGAGCAAAG-3′
*Runx2*	5′-AGATGATGACACTGCCACCTCTG-3′	5′-GGGATGAAATGCTTGGGAACT-3′
*COL I*	5′-CATCTCCCCTTCGTTTTTGA-3′	5′-CCAAATCCGATGTTTCTGCT-3′
*OC*	5′-TGTGAGCTCAATCCGGCATGT-3′	5′-CCGATAGGCCTCCTGAAGC-3′
*OPN*	5′-CAGGTCTGCGAAACTTCTTAG-3′	5′-CTCCATTGACTCGAACGACTC-3′

## Data Availability

Data supporting the findings of this study are available from the corresponding authors upon proper request.

## Supplementary Materials

The following supporting information can be downloaded at: https://www.mdpi.com/article/10.3390/ijms241713203/s1.
