# Supplementary material for: Novel Electroactive Mineralized Polyacrylonitrile/PEDOT:PSS Electrospun Nanofibers for Bone Repair Applications
Source: Int J Mol Sci. 2023 Aug 25;24(17):13203. doi: 10.3390/ijms241713203 (PMC10488027; doi:10.3390/ijms241713203)
Supplement: Supplementary file 1 [file ijms-24-13203-s001.zip › ijms-2543715-supplementary.pdf]

Supplementary Material

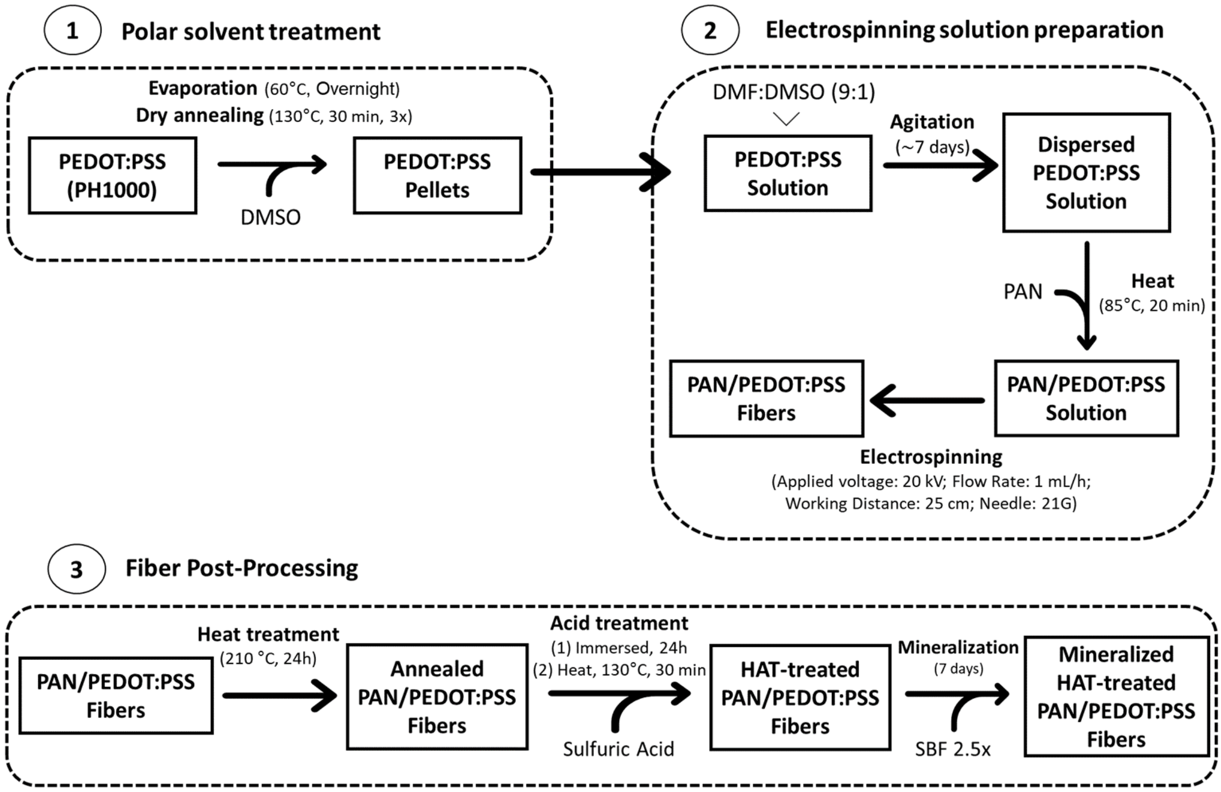

Scheme S1. Outline of the process used for preparing the PAN/PEDOT:PSS electrospinning solutions (1 and 2). Summary of the post-processing steps performed to obtain the mineralized HAT-treated PAN/PEDOT:PSS nanofibers (3).

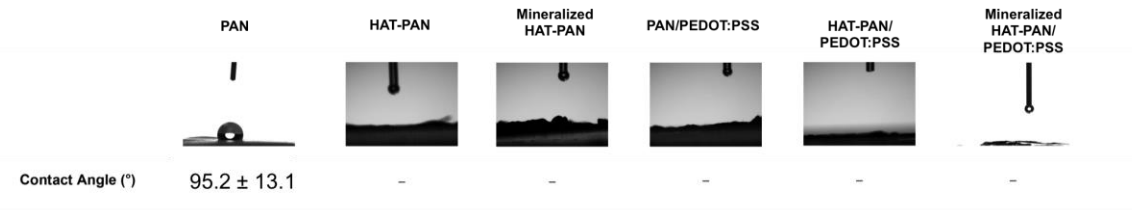

Figure S1. Sessile drop droplet profile and contact angle of the as-spun and HAT-treated PAN and PAN/PEDOT:PSS nanofibers as well as of mineralized HAT-treated PAN/PEDOT:PSS nanofibers. Seven different samples (n=7) were used in the analysis.

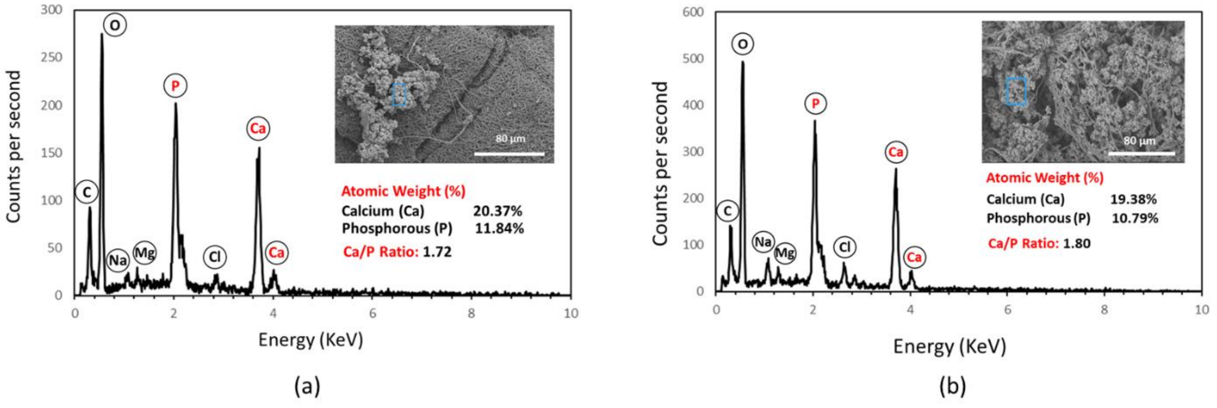

Figure S2. Elemental composition analysis (EDX) of the generated HAT-PAN (a) and HAT-PAN/PEDOT:PSS (b) nanofibers after 7 days incubation in SBF 2.5x. SEM images of the spots where EDX analysis was conducted are presented inside the corresponding EDX spectrograms. The atomic percentage (Atomic%) of calcium (Ca) and phosphorus (P) present in each sample (used in Ca/P ratio computation) is highlighted.

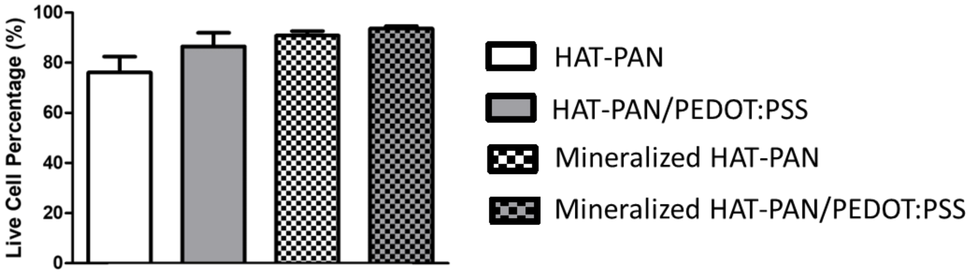

Figure S3. Estimated percentage (%) of viable osteoblast-like MG-63 cells on the mineralized and non-mineralized HAT-PAN and HAT-PAN/PEDOT:PSS nanofibers after 10 days of culture. Three different samples (n=3) per condition were used in the analysis.

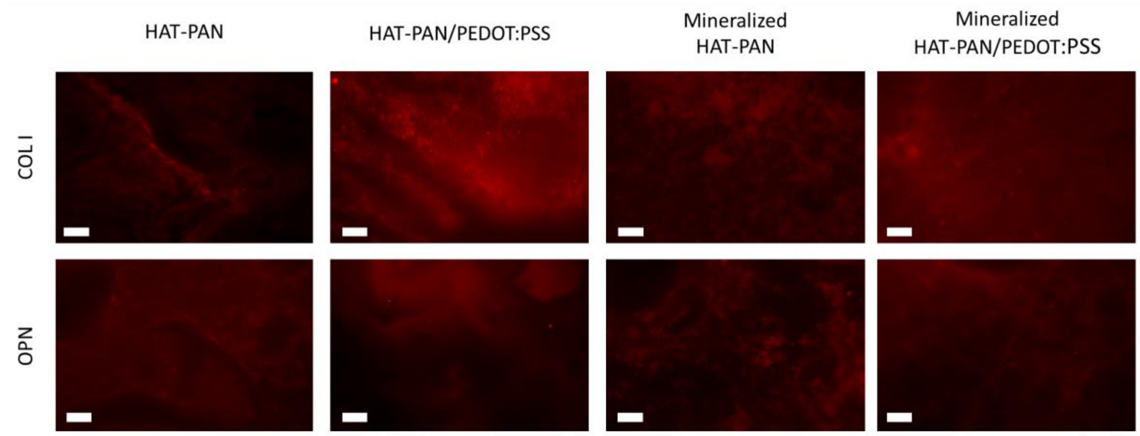

Figure S4. Immunofluorescence analysis to evaluate the presence of type I collagen (COL I) and osteopontin (OPN) on the electrospun scaffolds after 21 days of osteogenic differentiation. COL I and OPN antibodies stain collagen and osteopontin molecules red, respectively. Scale bar: 100 μm.

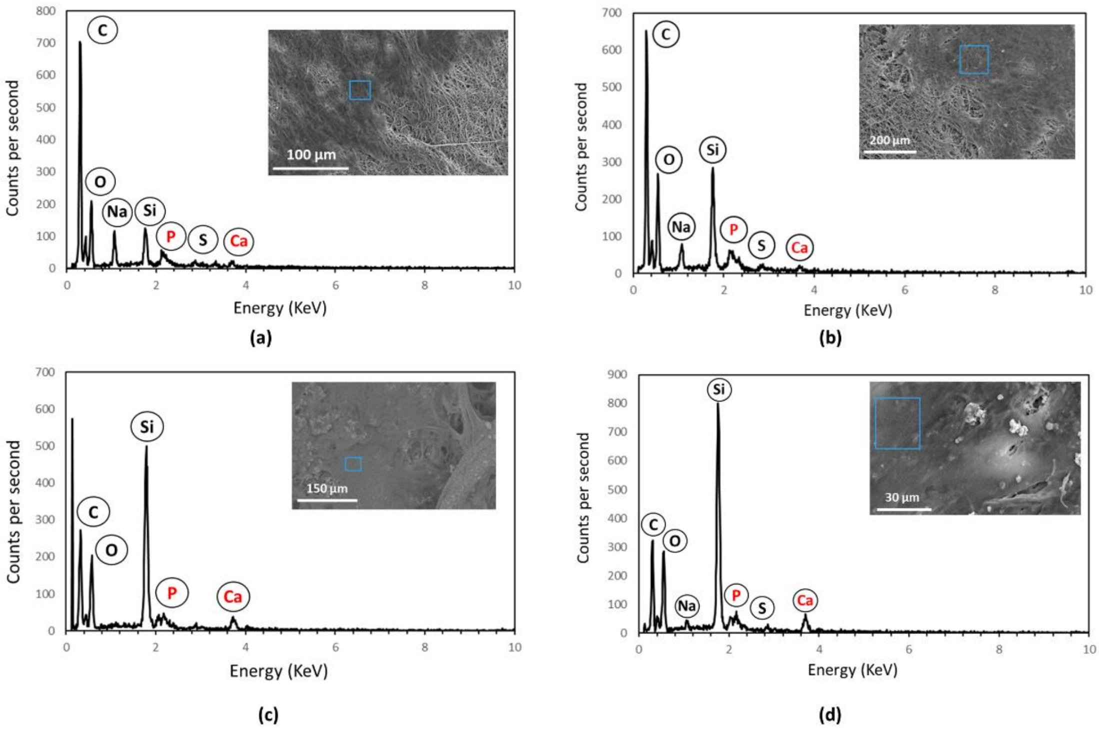

Figure S5. Elemental composition analysis (EDX) of the MSC-seeded HAT-PAN (a), HAT-PAN/PEDOT:PSS (b), mineralized HAT-PAN (c) and mineralized HAT-PAN/PEDOT:PSS (d) nanofibers after 21 days of osteogenic differentiation. SEM images of the spots where EDX analysis was conducted are presented inside the respective EDX spectrograms.
